# Headache

**Published:** 1909-11-20

**Authors:** 


### Diseases of Children.

### HEADACHE.

"De doloribus capitis; scandalo medicorum, difficulter removendo." Such were the words of Juncker, and they still remain true. In young children headache should never be neglected. It is a more significant sign in them than in older people, and may indicate the onset of acute or dangerous disease. Both in infants and young children it is quite frequent, and hardly receives the attention it deserves. It is a symptom of most febrile conditions, toxæmia, constitutional states, hygienic defects, nervous conditions, and various physical defects. The most important predisposing causes are an inherited weakness or instability of the nervous system, and that peculiarity of constitution known as the gouty diathesis. Probably the rheumatic diathesis is implicated in some cases. For instance, a boy of ten years had been subject to attacks of pain in the right frontal region for twelve months. The attacks were sufficiently severe to make him cry out and light-headed. They were associated with visual hallucinations. He saw people in the room, under the bed, and up the chimney. Each attack lasted for one to three days. There was no vomiting, no anæmia, and no cause could be ascertained. He came under treatment for chorea, and had a rheumatic family history. The gouty diathesis leads to irregular metabolism, and the consequent headache must be looked upon as toxæmic. Children of neurotic parentage, or themselves neurotic, are liable to headache on slight provocation, such as excitement, emotional disturbance, and active brain work. In some instances it is the result of educational pressure. Many an adult suffers from headaches as the result of premature strain on the brain during school life.

In the classification of headaches we must exclude "dolor capitis," pain due to changes in the scalp or bones, not inside the cranium. The three main groups are: (1) *Structural*, (2) *Hæmic*, (3) *Nervous*. Structural headaches are caused by concussion or contusions of the brain by falls and other injuries, inflammation of the meninges and of the cerebral tissues, meningeal cysts and growths on the dura mater, and intra-cerebral tumours. The pain is most severe when the nerve endings in the meninges are involved.

The hæmic causes include anæmia, hyperæmia, and altered blood states, namely, fevers, uræmia, etc.; the intestinal toxæmia of auto-intoxication, constipation, worms, and sluggish liver; poisons of lead, $CO_2$, bad atmosphere and drugs; and malaria. In some of these there is definite vascular engorgement, in others toxæmia arising from a variety of different affections. The nervous headaches include paroxysmal recurrent headache, not uncommon in childreh, and migraine; those due to noise and brain exhaustion; those of sympathetic origin, dependent on disorders elsewhere; others ascribed to reflex irritation, as from decayed or impacted teeth, errors of refraction, ear troubles and affections of the naso-pharynx. Many of the last group are more or less neuralgic, the irritation being that of some branch of the fifth nerve, and the skin area is tender. Though such a classification is useful, it must not be supposed that all headaches can be systematically labelled and pigeon-holed in one or other group. Many of them are due to more than one, often to several combined causes.

Headache is frequently due to impaired general health and anæmia from any cause, for this impairs the nutrition of the nerve cells. Its features are that it is relieved by recumbency, increased by the upright posture, and temporarily benefited by alcohol. Sometimes there is vertigo or nausea on rising, especially on getting up suddenly. Alimentary disorders act reflexly by setting up indigestion and constipation, or through the absorption of the toxic products of unsuitable or imperfectly digested food, dependent on some by-products of digestion which are imperfectly elaborated and act as poisons. The headache, due to the vitiated atmosphere of stuffy, ill-ventilated rooms, is partly the result of an excess of carbonic acid gas in the inspired air, and partly due to the inspiration of deleterious products given off from the lungs, skin, and clothes of others present. The excess of carbonic acid gas is probably of minor importance, for it is by no means common to find headaches in cases of cyanosis. Deficient oxygenation of the blood is a definite cause, however, in many respiratory troubles. Most important of these in children are adenoids and hyperæmia of the nasal mucous membrane. Pavor nocturnus, nervous irritability, and sluggishness of intellect are associated symptoms. Eye-strain is a frequent cause, and comes into play at the beginning of school life. It depends on errors of refraction, generally hypermetropia or astigmatism. The pain is most often situated in the occipital region. That caused by astigmatism may be bi-temporal or frontal.

The localisation of headache is a doubtful clue to its pathology. That of fever and toxæmia is generalised. Severe frontal headache with rise of temperature is common at the onset of a specific fever. Such a headache disappears with the onset of delirium, whereas that of organic disease persists.

Frontal headache is caused by anæmia, toxæmia, eye-strain, and affection of the naso-pharynx. Affections of the nose and frontal sinuses may cause pain in the supra-orbital or frontal region. Vertical headaches are due to anæmia, hysteria, epilepsy, and meningitis. Parietal ones are caused by ear troubles and carious teeth. Decayed molars may cause occipital headache. In inflammation of the tympanum, middle ear or Eustachian tube the pain is often referred to the external ear or its neighbourhood. It is rather a "dolor capitis" than a true headache. Occipital headaches may be set up by otitis media, epilepsy, meningitis, cervical caries, cerebellar disease, eye-strain, and pharyngitis. A localised headache is suggestive of tumour or abscess. It may sometimes be relieved by the simple remedies useful in other varieties, for the pain is produced by hyperæmia or irritation.

